# GC-MS Profiling, Vibriocidal, Antioxidant, Antibiofilm, and Anti-Quorum Sensing Properties of *Carum carvi* L. Essential Oil: In Vitro and In Silico Approaches

**DOI:** 10.3390/plants11081072

**Published:** 2022-04-14

**Authors:** Siwar Ghannay, Kaïss Aouadi, Adel Kadri, Mejdi Snoussi

**Affiliations:** 1 Department of Chemistry, College of Science, Qassim University, Buraidah 51452, Saudi Arabia; s.ghannay@qu.edu.sa (S.G.); k.aouadi@qu.edu.sa (K.A.); 2 Faculty of Sciences of Monastir, University of Monastir, Avenue of the Environment, Monastir 5019, Tunisia; 3 Department of Chemistry, Faculty of Science of Sfax, University of Sfax, BP1171, Sfax 3000, Tunisia; lukadel@yahoo.fr; 4 Faculty of Science and Arts in Baljurashi, Albaha University, P.O. Box 1988, Albaha 65527, Saudi Arabia; 5 Department of Biology, College of Science, Hail University, P.O. Box 2440, Hail 2440, Saudi Arabia; 6 Laboratory of Genetics, Biodiversity and Valorization of Bio-Resources (LR11ES41), Higher Institute of Biotechnology of Monastir, University of Monastir, Avenue Tahar Haddad, BP74, Monastir 5000, Tunisia

**Keywords:** volatile oil, *Carum carvi* L., antioxidant, *Vibrio* spp., biofilm, anti-quorum sensing, pharmacokinetics, molecular docking

## Abstract

The main objectives of the present study were to investigate anti-*Vibrio* spp., antibiofilms, and anti-quorum-sensing (anti-QS) properties of caraway essential oil in relation to their phytochemical composition. The results obtained show the identification of twelve compounds, with carvone (58.2%) and limonene (38.5%) being the main ones. The obtained essential oil (EO) is particularly active against all *Vibrio* spp. species, with bacteriostatic action against all tested strains (MBC/MIC ratio ≥ 4) and with inhibition zones with high diameters of growth, ranging from 8.66 ± 0.58 mm for *V. furnisii* ATCC 35016 to 37.33 ± 0.58 mm for *V. alginolyticus* ATCC 17749. Caraway essential oil (Carvone/limonene chemotype) exhibits antioxidant activities by using four tests (DPPH = 15 ± 0.23 mg/mL; reducing power = 7.8 ± 0.01 mg/mL; β-carotene = 3.9 ± 0.025 mg/mL; chelating power = 6.8 ± 0.05 mg/mL). This oil is particularly able to prevent cell-to-cell communication by inhibiting swarming motility, production of elastase and protease in *Pseudomonas aeruginosa* PAO1, and violacein production in *C. violaceum* in a concentration-dependent manner. A molecular docking approach shows good interaction of the identified bioactive molecules in caraway EO, with known target enzymes involved in antioxidant, antibacterial, and anti-QS activities having high binding energy. Overall, the obtained results highlight the possible use of caraway essential oil against pathogenic *Vibrio* species and to attenuate the secretion of virulence-related factors controlled by QS systems in Gram-negative bacteria. Therefore, this oil can be used by food industries to prevent biofilm formation on abiotic surfaces by *Vibrio* strains.

## 1. Introduction

Bacteria belonging to the genus *Vibrio* are natural hosts of the marine environment [1,2,3]. The ubiquity of these bacteria in the marine environment and the potential seriousness of these infections, especially for sensitive people, have drawn attention to these microorganisms [4,5]. Ultra-recent advances in understandings of bacterial behavior have shown the existence of a cell-to-cell communication mechanism called quorum sensing, which is involved in the regulation of social behavior and expression of virulence factors involved in their pathogenicity [6]. Plant-derived molecules, due to their wide spectrum of biological activities and especially due to their antimicrobial and antioxidant properties, have long been well recognized in medicine and pharmacy [7], such as antioxidant [8,9,10,11,12,13], antimicrobial [10,11,12,13], cytotoxicity [12,13], anti-acetylcholinesterase [12,13], and antidiabetic [13] properties as well as anti-inflammatory [9] and preventive effects on the cardiac remodeling process [14]. Thus, essential oils (EOs) from many plants have been well-used for hundreds of years to fight an infinite number of pathogens, including bacteria, viruses, and fungi [14,15,16]. Recently, great attention has been paid to the efficacy and safety of nanomedicine based on natural products in preclinical models of various diseases as well as for other applications [17,18,19,20,21]. EO also has anti-QS activity and targets the QS system via three pathways, either by the breakdown of the synthesis of signal molecules or their degradation or by blocking their receptors. Biofilm formation is controlled by QS mechanisms, which makes biofilm formation and QS two central and interconnected features of the social life of bacteria. Indeed, the fight against biofilms can be defined according to two main axes: preventing the formation of biofilms and destroying them when they are already present [22].

*Carum carvi* L., or caraway, belongs to the Apiaceae family, which is a biennial plant species with fine and grooved stems. The leaves are oblong, and its white flowers, sometimes pink, appear in umbels. Its seeds are 5 mm in measurement. Its size does not exceed 60/75 cm in height at most. Carvi is made up of D-carvone-rich essential oil, together with fatty oils and polysaccharides [23]. The fruits are used in traditional medicine as a carminative, and it is added to young children’s dishes to help digestion and is added to food products in rye bread for its taste and flavor [24]. Caraway possesses several pharmacological properties, including antispasmodic, emmenagogue, expectorant, galactagogue, stimulant, stomachic, and tonic properties [24]. Moreover, carvi EO has been proved for its cancer chemopreventive effects against colon premalignant injuries induced by dimethylhydrazine [25]. The antimicrobial, antioxidant, anti-acetylcholinesterase, and antidiabetic activities of caraway essential oil have also been reported in our laboratory [16].

Hence, the aim of this work was to study the chemical composition of caraway seeds commonly used in the Saudi kitchen to prepare fish and shellfish dishes. Antibacterial activity has been tested against several pathogenic *Vibrio* spp. strains. The ability of the obtained volatile oil to scavenge reactive oxygen species in vitro using different assays has been assessed. An in silico approach was performed in order to elucidate its physicochemical properties, pharmacokinetic properties, drug-likeness, and toxicity prediction of the identified bioactive compounds in caraway essential oil.

## 2. Results

### 2.1. Chemical Profile of C. carvi EO

The yield of extraction was about 3.52% (*v*/*w*) based on dry weight. Twelve phytoconstituents representing 98.7% of the total oil composition were identified (Table 1).

**Figure 1:**
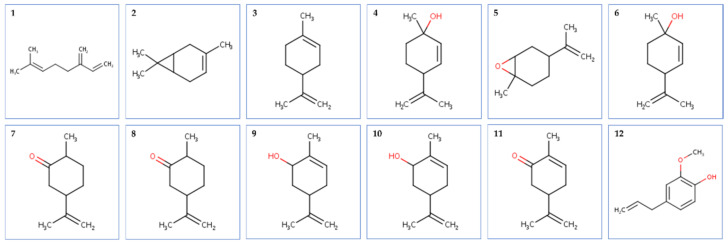
Structure of phytocompounds identified by GC-MS technique from caraway essential oil. Legend: (**1**): Myrcene; (**2**): D-3-carene; (**3**): Limonene; (**4**): *trans*-*p*-mentha-2.8-dien-1-ol; (**5**): *cis*-limonene oxide; (**6**): *cis*-*p*-mentha-2.8-dien-1-ol; (**7**): *cis*-dihydrocarvone; (**8**): *trans*-dihydrocarvone; (**9**): *trans*-carveol; (**10**): *cis*-carveol; (**11**): Carvone; (**12**): Eugenol.

The tested oil was dominated by oxygenated monoterpenes (59.6%), followed by monoterpene hydrocarbons (39%) and phenylpropanoids (0.1%). Carvone (58.2%) and limonene (38.5%) were the main compounds identified. The chemical structures of the identified compounds are represented in Figure 1.

### 2.2. Antioxidant Activities Screening

The antioxidant activity of caraway essential oil examined using four different assays (DPPH, reducing power, β-carotene, and chelating power) is outlined in Table 2. According to the results of the DPPH free radical scavenging assay, caraway essential oil exhibits potent antioxidant effects with an IC_50_ value of 15 ± 0.23 mg/mL, which is significantly (*p* < 0.05) lower than that of BHT and ascorbic acid, used as standards.

Interestingly, the results show that caraway essential oil displays a significantly higher redox property (2.95–3.2 times) towards the reducing power test when compared to the commercial standards, BHT and ascorbic acid. Moreover, the chelating power of caraway essential oil is significantly (*p* < 0.05) higher (4.7 times) than that of EDTA (IC_50_ = 32.50 ± 1.32 mg/mL), used as a positive control. A comparison of the capacity of caraway essential oil to inhibit linoleic acid oxidation to that of BHT points out very similar results (IC_50_ = 3.9 ± 0.025 vs. 4.60 ± 1.60) with no significant difference (*p* > 0.05).

### 2.3. Antimicrobial Activity

#### 2.3.1. Vibriocidal Activities

The antimicrobial activity of caraway essential oil was qualitatively and quantitatively assessed by the presence or absence of an inhibition zone, MIC, and MBC values (Table 3). The results obtained from the disc diffusion method indicate that caraway chemotype (Carvone/limonene) tested at 10 mg/disc exhibited antimicrobial activity against all *Vibrio* spp. with various degrees of antimicrobial activity depending on the strain tested.

In fact, the obtained EO from caraway seeds was tested against a large collection of *Vibrio* spp. strains, including pathogenic ones isolated from diseased reared fish (*Dicentrarchus labrax* and *Sparus aurata*), from *Mytilus edulis*, and from seawater. At 10 mg/disc, carvone-limonene rich oil was able to inhibit the growth of all *Vibrio* ssp. strains with different degrees. Indeed, *V. alginolyticus* strains were the most sensitive ones with growth inhibition zones (GIZs) ranging from 11.67 ± 0.58 mm to 37.33 ± 0.58 mm. Similarly, *V. parahaemolyticus* strains were also sensitive to the tested oil with GIZs ranging from 11.67 ± 0.58 mm for *V. parahaemolyticus* isolated from mussels to 25.33 ± 0.58 mm for *V. parahaemolyticus* isolated from seawater. In addition, a low concentration of caraway EO was needed to kill almost all tested strains with MBC values ranging from 5.781 mg/mL to 23.125 mg/mL. Using the MBC/MIC ratio, the tested oil exhibited bacteriostatic activity against all tested bacteria (MBC/MIC ratio > 4). All these data are summarized in Table 3.

#### 2.3.2. Biofilm Inhibition and Eradication

Vibrionaceae members are mainly aquatic bacteria present in different forms: those that are free planktonic in oceans or estuaries, those that are associated with biotic or abiotic surfaces in biofilms, and finally those that colonize marine animals. Caraway volatile oil was tested for its ability to prevent and eradicate the biofilms formed by four *Vibrio* species, including *V. cholerae*, *V. vulnificus*, *V. parahaemolyticus*, and *V. alginolyticus* by using the XTT technique.

**Figure 2:**
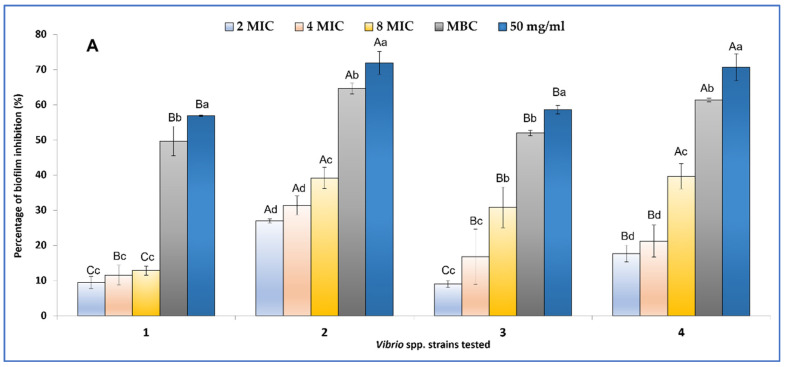

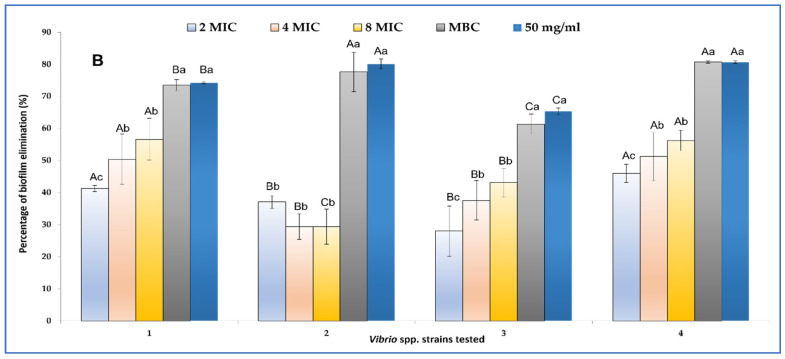
Effects of different concentrations of *C. carvi* EO on biofilm formation (**A**) and eradication (**B**) expressed as percentages of inhibition evaluated by the XTT technique: *V. alginolyticus* ATCC 33787 (**1**, MIC value = 0.022 mg/mL)*, V. parahaemolyticus* ATCC 17802 (**2**, MIC value = 0.022 mg/mL), *V. vulnificus* ATCC 27962 (**3**, MIC value = 0.022 mg/mL)*,* and *V. cholerae* ATCC 9459 (**4**). Errors bars represent standard deviation. Values are the average of at least three independent determinations. Means followed by the same letters are not significantly different at *p* < 0.05 based on Duncan’s multiple range test. Small letters are used to compare different concentrations within the same strain, and capital letters are used to compare means between the same concentrations between strains.

Results obtained show that biofilm formation (Figure 2A) was inhibited by 9.46 ± 1.67% for *V. alginolyticus* ATCC 33787, by 26.95 ± 0.65% for *V. parahaemolyticus* ATCC 17802, by 9.02 ± 1.03% for *V. vulnificus* ATCC 27962, and by 17.66 ± 2.39% for *V. cholerae* ATCC 9459 at 0.044 mg/mL (2MIC value). At sub-MIC concentrations (50 mg/mL), the formation of biofilms on 96-well plates was inhibited by 71.90 ± 3.26% for *V. parahaemolyticus* ATCC 17802 and by 70.66 ± 3.84% for *V. cholerae* ATCC 9459.

Interestingly, the tested caraway essential oil was able to eradicate more than 50% of preformed *V. alginolyticus* ATCC 3378 and *V. cholerae* ATCC 9459 biofilms at 0.088 mg/mL. MBC values are needed to eradicate 50% of biofilms formed by *V. parahaemolyticus* ATCC 17,802 and *V. vulnificus* ATCC 27,962 (Figure 2B).

#### 2.3.3. Anti-QS Activity

Caraway essential oil was also tested against virulence-related properties controlled by the QS system in *C. violaceum* (violacein production) and *P. aeruginosa* PAO1 (swarming, elastase, and protease production). In fact, caraway essential oil and its main compound (Carvone) were able to inhibit the swarming activity of *P. aeruginosa* PAO1 starter strain on LB-0.5% in a concentration-dependent manner (Table 4). The motility of this bacterium was reduced by 67.90% at 0.05 mg/mL for caraway essential oil and by 79.01% at 2.5 mg/mL. The main compound, carvone, was able to inhibit the motility of *P. aeruginosa* PAO1 by 71% at 0.05 mg/mL and by more than 79.62% at 2.5 mg/mL of the same essential oil.

**Figure 3:**
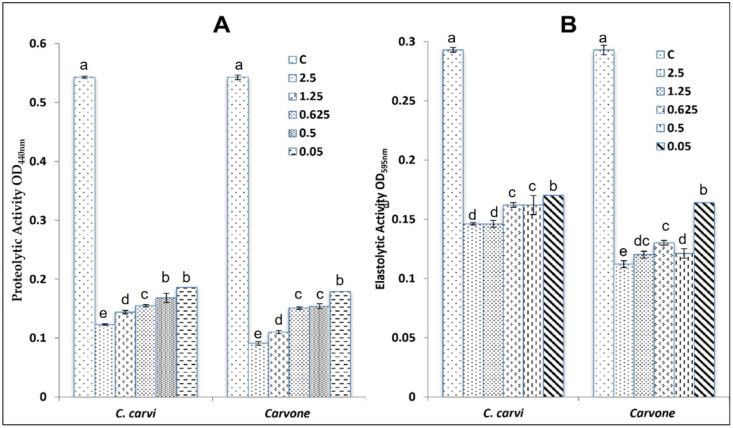
Effect of caraway essential oil and its main compound (Carvone) tested at different concentrations on *P. aeruginosa* PAO1 (virulence-related properties controlled by quorum-sensing system: (**A**) proteolytic activity; (**B**) elastolytic activity). Values are the average of at least three independent determinations. Means followed by the same letters are not significantly different at *p* < 0.05 based on Duncan’s multiple range test.

Production of elastase and protease by the PAO1 strain was also inhibited by caraway essential oil, as shown in Figure 3. In fact, proteolytic activity was reduced by 65.74% at 0.05 mg/mL for caraway essential oil and by 67.03% for carvone. At high concentrations (2.5 mg/mL), this oil inhibited the production of protease enzymes in the PAO1 strain by 77.34% and by 83.24% for carvone. Elastase production was also affected by caraway essential oil and carvone in a concentration manner. The highest inhibition was recorded at 2.5 mg/mL and ranged from 50.17% for the oil to 61.77% for its main compound (carvone).

**Figure 4:**
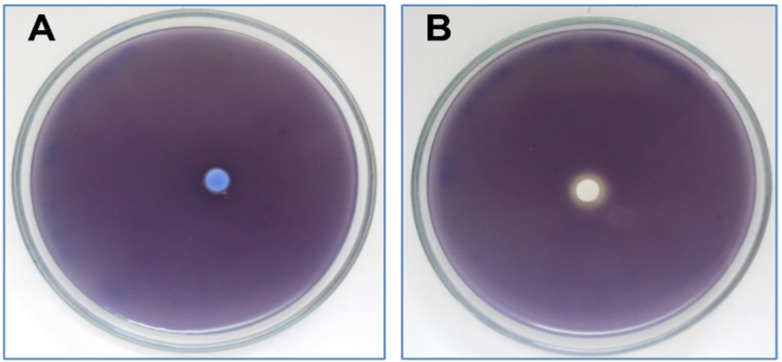
(**A**) Effect of caraway essential oil on violacein production by *C. violaceum* CV026 tested on Lauria–Bertani agar as compared to limonene at 2 mg/disc (**B**).

This oil has the ability to inhibit the production of violacein by two different techniques: on LB agar Petri dishes using the mutant strain CV026 and on microtitre plates by using *C. violaceum*-type strains (ATCC 12472). The results obtained show that the tested oil (Figure 4A) and its major compound (carvone) were unable to inhibit the production of violacein at 2 mg/disc, whereas limonene at 2 mg/disc (Figure 4B) slightly inhibited violacein production (Inhibition zone = 2 mm).

As depicted in Table 5, caraway essential oil inhibited the production of violacein by *C. violaceum* wild type (ATCC 12472) in a concentration-dependent manner. In fact, at an MIC value of 10 mg/mL, the percentage of violacein production in 96-well plates was about 47.57 ± 3.7%. At low MIC values (MIC/32 = 0.312 mg/mL), the production of this pigment was inhibited by 25.28 ± 4.3%. All these data are summarized in Table 5.

### 2.4. Molecular Docking Analysis

#### 2.4.1. Molecular Docking Antimicrobial Receptor Proteins

DNA gyrase is an enzyme belonging to a member of bacterial topoisomerase, which, by introducing transient breaks to both DNA strands, can control the topology of DNA during transcription, replication, and recombination. Therefore, this enzyme is essential for bacterial survival and can mainly be exploited as an antibacterial drug target. Here, we attempted to investigate the binding pattern of the most relevant phytocompounds. Molecular docking of *trans*-dihydrocarvone, eugenol, and *trans*-carveol, the top three compounds that have the best binding affinity, were performed to identify their binding sites on the structures of *S. aureus* tyrosyl-tRNA synthetase (PDB ID, 1JIJ) and topoisomerase II DNA gyrase (PDB ID, 2XCT) proteins. In fact, *trans*-Dihydrocarvone, *trans*-carveol, and eugenol were found to be the most stable complexes with tyrosyl-tRNA synthetase possessing a binding energy of −6.3 kcal/mol, −6.4 kcal/mol, and −6.3 kcal/mol, respectively.

**Figure 5:**
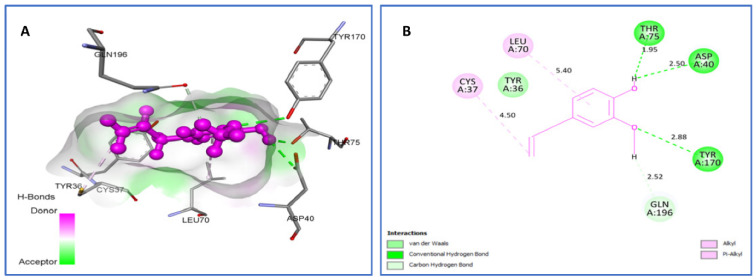

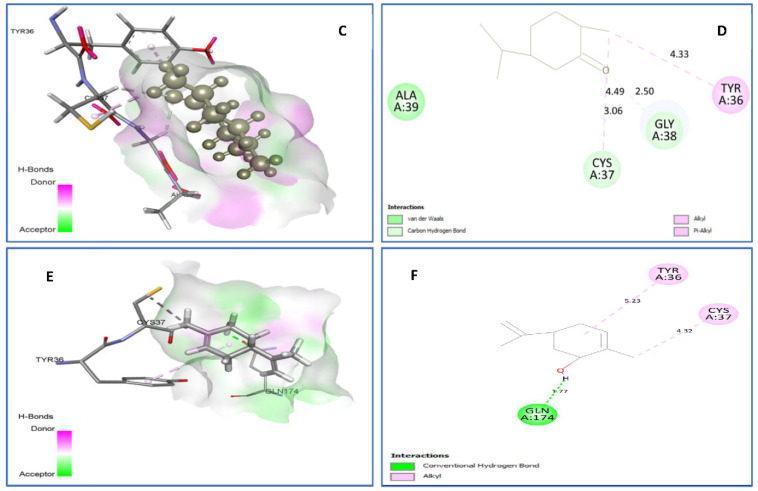
Two-dimensional (2D) and three-dimensional (3D) residual interactions network of eugenol (**A**,**B**), *trans*-dihydrocarvone (**C**,**D**), and *trans*-carveol (**E**,**F**) with the active site of tyrosyl-tRNA synthetase (PDB Id: 1JIJ) enzyme.

As shown in Figure 5, *trans*-Dihydrocarvone forms van der Waals interactions with Ala39; C-H bonds with Cys37(3.06) and Gly38 (2.50); and Alkyl/Pi-Alkyl with Tyr36 (4.33) and Cys37 (4.49) amino acids. *Trans*-Carveol binds to active sites via hydrophobic interactions with Cys37, Ala39, and Tyr36 residues; however, eugenol establishes van der Waals bonding with Tyr36; H bonds with Asp40 (2.50), Thr75 (1.95), and Tyr170 (2.88); C-H bonds with Gln196 (2.52); and Alkyl/Pi-Alkyl with Cys37 (4.50), Leu70 (5.40).

**Figure 6:**
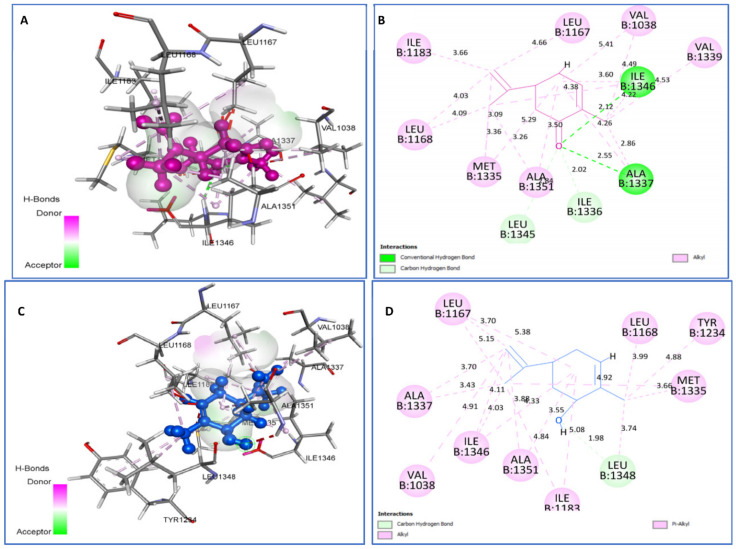
Two-dimensional (2D) and three-dimensional (3D) residual interactions network of carvone (**A**,**B**) and *cis*-carveol (**C**,**D**) with the active site of topoisomerase II DNA gyrase (PDB Id: 2XCT) enzyme.

The best-docked natural ligands for topoisomerase II DNA gyrase were found to be cis-carveol and carvone, with the same binding energies of −5.3 kcal/mol. Cis-Carveol forms C-H bonds with Leu1348 and Alkyl/Pi-Alkyl with Ala1351, Ile1346, Leu1168, Met1335, Leu1348, Leu1167, Ile1183, Ile1346, Val1038, and Tyr1234. However, carvone binds to 2XCT by two H bonds, Ala1337 (2.55) and Ile1346 (2.12); one C-H bond with Ile1336 and Leu1345; and Alkyl interactions with Val1038, Ala1337, Met1335, Ile1346, Leu1168, Val1339, and Leu1167 residues (Figure 6).

#### 2.4.2. Molecular Docking against Antioxidant Receptor Proteins

**Figure 7:**
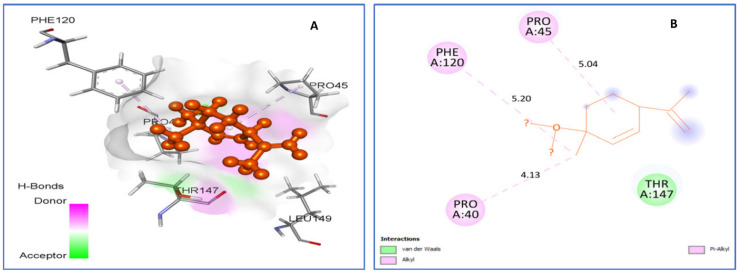
Two-dimensional (2D) and three-dimensional (3D) residual interactions network of *trans*-*p*-mentha-2.8-dien-1-ol (**A**,**B**) with the active site of Human peroxiredoxin 5 (PDB ID: 1HD2) enzyme.

Human peroxiredoxin 5, located mainly in mitochondria, peroxisomes, and cytosol, is implicated in antioxidant protective mechanisms as well as in signal transduction in cells, and thus it is a potential target for evaluation of antioxidant activity. *Trans*-*p*-mentha-2.8-dien-1-ol (Figure 7) was found to be best-docked to human peroxiredoxin 5 with a binding energy of −5.2 kcal/mol. It formed hydrophobic interactions with residues Thr147, Pro40 (4.13), Pro45 (5.04), and Phe120 (5.20).

#### 2.4.3. Molecular Docking against QS Receptor Proteins

**Figure 8:**
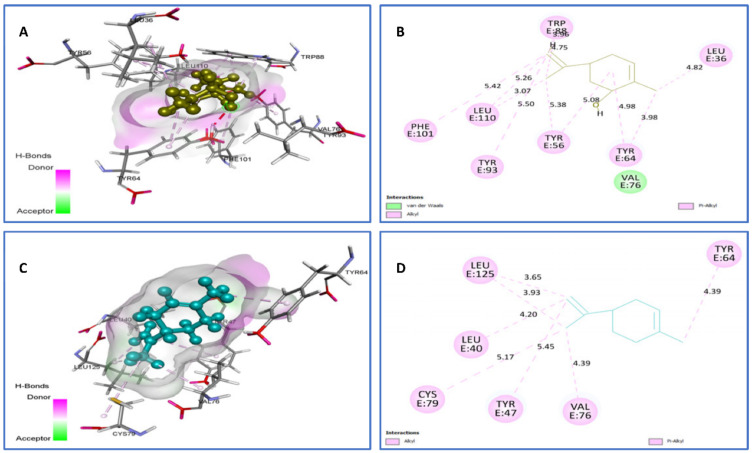
Two-dimensional (2D) and three-dimensional (3D) residual interactions network of *trans*-carveol (**A**,**B**) and limonene (**C**,**D**) with the active site of LasR enzyme (PDB ID, 2UV0).

To obtain insight of the possibility of binding interactions between identified phytocompounds and QS receptors, docking studies were performed. The crystal structures of QS signal receptors LasR (PDB ID, 2UV0) and (PDB ID: 3IX3), from *P. aeruginosa* as a key regulator of *P. aeruginosa* pathogenesis, and CviR (PDB ID, 3QP1), from *C. violaceum* ATCC 12472, were used for docking analysis. Limonene and *trans*-carveol were selected as the top natural ligands against LasR (PDB ID 2UV0, Figure 8) because of their good binding energies within the active site, which are −7.4 kcal/mol and −7.5 kcal/mol, respectively. Their contacts suggest that limonene forms van der Waals interactions with Val76 and Alkyl/Pi-Alkyl with Tyr93 (5.50), Tyr56 (5.08) (5.38), Tyr64 (3.98) (4.98), Leu36 (4.82), Leu110 (3.07) (5.26) (5.42), and Trp88 (3.96) (4.75), all of which are reported in the literature based on X-ray structural observations (Figure 8). In addition, the *trans*-carveol-2UV0 complex involves the following interactions: Alkyl/Pi-Alkyl with Cys79 (5.17), Tyr47 (5.45), Val76 4.39, Leu40 (4.20), Leu125 (3.65) (3.93), and Tyr64 (4.93).

**Figure 9:**
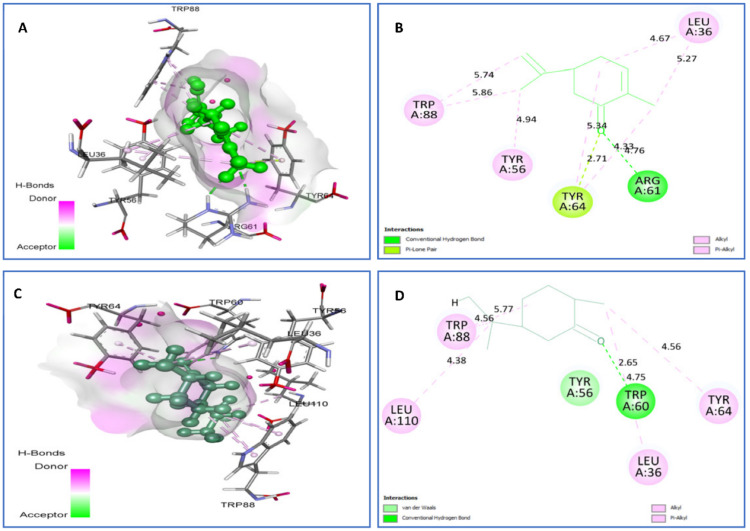
Two-dimensional (2D) and three-dimensional (3D) residual interactions network of carvone (**A**,**B**) and *trans*-dihydrocarvone (**C**,**D**) with the active site of LasR enzyme (PDB ID, 3IX3).

Towards LasR (PDB ID: 3IX3; Figure 9), carvone and *trans*-dihydrocarvone were also selected as the top natural ligands. Carvone was found to interact with the following 3IX3 residues: H-bonds with Arg61 (2.71), P-Lone Pair with Tyr64 (2.71), and Alkyl/Pi-Alkyl with Leu36 (4.67) (5.27), Tyr56 (4.94), Tyr64 (4.33) (4.76), and Trp88 (5.74) (5.86). However, *trans*-dihydrocarvone was able to establish H bonds with Trp60 (2.65), van der Waals contacts with Tyr56, and Alkyl/Pi-Alkyl with Leu36 (4.75), Tyr64 (4.56), Trp88 (4.56) (5.71), and Leu110 (4.38).

**Figure 10:**
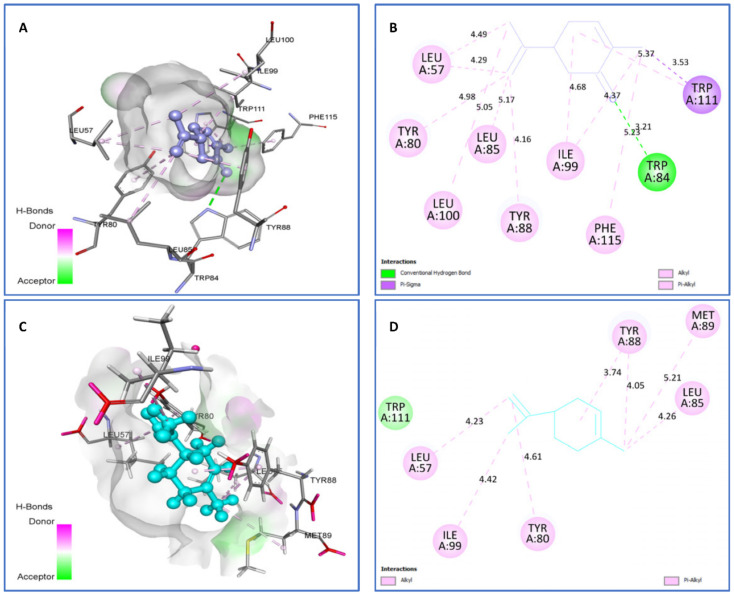
Two-dimensional (2D) and three-dimensional (3D) residual interactions network of carvone (**A**,**B**), and limonene (**C**,**D**) with the active site of CviR enzyme (PDB ID, 3QP1).

On the other hand, towards the CviR protein (Figure 10), limonene (−7.4 kcal/mol) and carvone (−7.5 kcal/mol) were highly bound ligands, with limonene showing van der Waals interactions with Trp111 and Alkyl/Pi-Alkyl with Ile99 (4.42), Tyr80 (4.61), Leu57 (4.23), Leu85 (4.26), Met89 (5.21), and Tyr88 (3.74) (5.21). However, carvone displayed one hydrogen bonding interaction with Trp84 (3.21), one Pi-Sigma with Trp111 (3.53), and several Alkyl/Pi-Alkyl with Leu100 (5.05), Tyr80 (4.98), Leu57 (4.29) (4.49), Leu85 (5.17), Tyr88 (4.16), Ile99 (4.37) (4.68), Phe115 (5.23), and Trp111 (5.37) amino acid residues.

Table 6 summarizes the best-identified bioactive compounds in caraway essential oil with all target proteins, their interacting residues, and binding energies (Kcal/mol).

### 2.5. ADMET Analysis

The study of the disposition of a drug molecule within an organism is an indispensable part of drug discovery based on the assessment of its pharmacokinetic properties, named Absorption, Distribution, Metabolism, Excretion, and Toxicity (ADMET) [23,24,25]. The ADMET properties, as derived from the admetSAR tool, reveal that all identified compounds have a good Human Intestinal Absorption (HIA) score, meaning that they are well-absorbed in the intestinal tract upon oral administration. They were found to easily penetrate to Caco-2 and are promoted to be potential substrates and inhibitors for P-glycoprotein (P-gp), which effluxes drugs and various compounds to undergo further metabolism and clearance, and they are thought to cross the blood–brain barrier (BBB) easily. In terms of metabolism, we found that all analogues are non-substrates and non-inhibitors of any CYP450 isoenzymes (with some exception for compound **5**), suggesting that they do not obstruct the biotransformation of drugs metabolized by the CYP450 enzyme. The toxicity profile was predicted through different parameters; the two compounds were identified as non-inhibitors of hERG (human-Ether-a-go-go-related), which is a gene that encodes the protein channel that potassium uses to contribute to heart rate activity, meaning that they do not disrupt the heart. Based on the Ames test, all compounds do not show any mutagenic effects. Moreover, they are not carcinogenic (except myrcene), and they are without any hepatotoxic effect. The acute toxicity test revealed that they are unlikely to present acute hazards (Table 7).

## 3. Discussion

Spices such as caraway (*C. carvi* L.) are widely used in Saudi Arabia to spice and aromatize fish and shellfish dishes. These plant species can be promising sources of phytochemical compounds active against *Vibrio* spp. pathogenic strains associated with seafood products. Therefore, the main objectives of the present study were to study the anti-*Vibrio* spp. activity of its EO in relation with its phytochemical composition. Our study revealed the identification of carvone (58.2%) and limonene (38.5%) as its main compounds. These percentages are in accordance with those reported by the European Pharmacopoeia, which defines that caraway essential oil must contain (50.0–65.0%) of carvone (30.0–45.0%) of limonene, (0.1–1.0%) b-myrcene, maximum (2.5%) *trans*-dihydrocarvone and a maximum of 2.5% for *trans*-carveol [26]. Additionally, it has been well documented that these two compounds are the main chemotype constituents of caraway essential oil from Tunisia, France, Greece, Ukraine, Moldova, Austria, and Norway, ranging from 38.4% to 45.9% [27].

The tested carvone/limonene chemotype of caraway essential oil is active against all *Vibrio* spp. with high growth inhibition zones and low MIC and MBC values. In fact, we reported the effectiveness of many plants species against the same strains tested, including *Mentha longifolia*, *M. pulegium*, *Eugenia caryophyllata*, *Thymus vulgaris* and *Rosmarinus officinalis Cuminum cyminum* L., *M. spicata* [28], *Elettaria cardamomum* [29], *Petroselinum crispum* and *Ocimum basilicum* [30], and *Allium roseum var. odoratissimum* [31].

In addition, thymol and carvacrol-rich *Lippia berlandieri* Schauer EO from Mexico (48% thymol/23% carvacrol and 25% thymol/40% carvacrol, respectively) are able to inhibit the growth of *V. alginolyticus*, *V. parahaemolyticus*, and *V. vulnificus* in shrimps [32]. In 2017, Partovi and colleagues [33] reported that essential oil extracted from *Artemisia absinthium*, *Zataria multiflora* Boiss., *Pulicaria gnaphalodes*, *Trachyspermum ammi,* and *C. cyminum* are highly active against pathogenic *V. parahaemolyticus* strains as compared to non-pathogenic ones with high diameters of growth inhibition zones, ranging from 23 ± 2.47 mm to 31 ± 1.41 mm for *V. parahaemolyticus* ATCC 43996 and from 15 ± 0.00 mm to 24 ± 1.41 mm for *V. parahaemolyticus* ATCC 17802. They also reported that low concentrations of these EOs are needed to inhibit the growth of these two *V. parahaemolyticus* ATCC strains (MIC values range from 0.025 to >3%, *v*/*v*), and MBC values range from 0.05 to >3% (*v*/*v*) [33].

All these differences in the effectiveness of *Vibrio* spp. for EO can be mainly attributed to the main compositions of the tested oils, the strains used, and the techniques applied. In fact, we mention the contributions of minor compounds, especially dihydrocarvone, eugenol, and *trans*-carveol as predicted by molecular docking, in our study. Dihydrocarvone as a monoterpene ketone has been illustrated for its antimicrobial activity as a potential growth inhibitor of yeast fungi such as *Saccharomyces cerevisiae, C. albicans*, and *Cryptococcus neoformans* [34].

The tested caraway essential oil also exhibited good antioxidant activity using four different assays (DPPH, reducing power, β-carotene, and chelating power) as compared to the standard molecules used (BHT, ascorbic acid, and EDTA). In fact, previous reports outline the antioxidant properties of caraway harvested from different ecotypes [16]. Additionally, it is appropriate to highlight here that the mechanism of EO action or its individual chemical constituents mainly depend on their chemical nature or chemical composition. Since antioxidant compounds can mitigate oxidative stress because of their scavenging capacity and/or their reducing power, research into powerful new antioxidants from plants has gained momentum in recent times. As has been shown, the antioxidant activity of *C. carvi* EO is likely due to the highly oxygenated mono-terpene content for which carvone is a major compound (58.2%). In addition, carvone (2-methyl-5-(1-methylethenyl)-2-cyclohexen-1-one, C_10_H_14_O), with its two enantiomeric forms, (−)-carvone and (+)-carvone, is an oxygenated monoterpene possessing a greater capacity to capture free radicals and to reduce power, and it has wide applicability in the food, beverage, and cosmetic industries. Our results corroborate very well with those of Hajlaoui et al. [16].

Our results show that the tested caraway essential oil (chemotype carvone/limonene) is able to inhibit/eradicate *Vibrio* spp. biofilm and to interfere with quorum-sensing systems in both *P. aeruginosa* PAO1 and *C. violaceum* starter strains. In fact, concentrations as low as 2MIC values (about 0.044 mg/mL) of this EO are able to inhibit biofilm formation by four *Vibrio* species, including *V. alginolyticus* ATCC 33787, *V. parahaemolyticus* ATCC 17802, *V. vulnificus* ATCC 27962, and *V. cholerae* ATCC 9459, with a percentage of biofilm inhibition ranging from 9.46 ± 1.67% to 26.95 ± 0.65%. In addition, the tested oil exhibits the ability to eradicate the biofilms formed by these *Vibrio* by 50% at 0.088 mg/mL. Similar results were reported by our team and other researchers who demonstrated the use of EO as a good alternative to *Vibrio* spp. biofilm formation and eradication [30,35,36]. In fact, EOs from *P. crispum* (Chemotype 1,3,8-p-menthatriene/β-phellandrene) and *O. basilicum* (Chemotype linalool/(E)-methylcinnamate) are able to inhibit and eradicate mature biofilms formed by the same strains at low concentrations. Similarly, *M. spicata* EO (Chemotype carvone/limonene) is able to inhibit biofilm formation by 11.5% and 11.6% for *V. alginolyticus* ATCC 33787, and by 28% and 40% for *V. vulnificus* ATCC 27562 at 0.046 and 0.092 mg/mL, respectively. The same EOs are able to eradicate more than 50% of preformed *V. cholerae* ATCC 9459 and *V. alginolyticus* ATCC 3378 biofilms at 0.092 mg/mL. In 2019, Mendes and colleagues [37] reported that *Protium heptaphyllum* EO (chemotype β-phellandrene/p-cymene) is able to inhibit biofilm formation by *V. parahaemolyticus* at 4 mg/mL due to increases in cell permeability causing the leakage of intracellular components and electrolytes. More recently, Mizan and colleagues [36] reported that clove, thyme, and garlic EOs are able to decrease colony count in biofilms formed on stainless-steel coupons by 0.48, 1.18, and 1.18 log CFU/cm^2^ at 1xMIC and by 3.60, 4.20, and 2.60 log CFU/cm^2^ at 8xMIC, respectively.

It has been demonstrated that many EOs can interfere with cell-to-cell communication by regulating virulence factors in many bacteria, including *P. aeruginosa* PAO1 and *C. violaceum* [38,39,40,41]. Our results show that caraway essential oil is able to inhibit the motility of *P. aeruginosa* PAO1 and to decrease the production of elastase and proteases at low concentrations. At MIC values, this EO and its main compound (carvone) are able to inhibit production of violacein by 25.28 ± 4.3%. By direct contact on Lauria–Bertani agar plates, no inhibition was recorded for both caraway essential oil and carvone. In fact, many EOs and phytochemical compounds are described for their abilities to decreases the production of virulence-related properties controlled by the quorum-sensing system in Gram-negative bacteria in a concentration-dependent manner [42,43,44,45]. These activities can be explicated by the effect of carvone (main compound identified in caraway essential oil) as a natural monoterpenoid with a high ability to control biofilm formation elimination by 80% at 60 to 70 µg/mL and violacein production in *C. violaceum* ATCC 12472 at the same concentration [46]. Similarly, it has been demonstrated that limonene is able to inhibit biofilm formation by *P. aeruginosa* ATCC 27853 and *P. aeruginosa* HT5 with different magnitudes and to decrease production of elastase enzymes at concentrations ranging from 0.1 to 4 mg/mL and ranging from (75% to 52%) to (80% to 66%), respectively [47]. Additionally, Luciardi and colleagues [48] reported that the attenuation of swarming, pyocyanin, and elastase production in the *P. aeruginosa* strain by limonene pure compounds is enhanced by compounds identified in *Citrus* limon oil. This result points out that biological activities of essential oils can be explicated by the synergism between single compounds identified [49].

Regarding docking, these findings corroborate well with our previous work, in which viridiflorol, methyleugenol, isocembrol, eugenol, α-selinene, and β-caryophyllene oxide [50], as well as 1,3-di-O-caffeoyquinic acid, *p*-coumaric acid, *trans*-ferulic acid, naringin, rosmarinic acid, rutin, salviolinic acid, 4,5-di-O-caffeoyquinic acid, apegen-in-7-o-glucoside, quercetrin (quercetin-3-o-rhamonoside), and cirsiliol [51], share the same amino acids when interacting with human peroxiredoxin 5 (1HD2). Moreover, Cys47, Thr44, Gly46, Thr147, Pro40, Pro45, Phe120, Arg127, and Leu149 are the main contributors in the stabilizing of the complex ascorbic acid-1HD2. Moreover, by analyzing the crystal structure of human peroxiredoxin 5 (1HD2), our results corroborate also with those of Noumi et al. [52], in which they docked the identified phytocompounds from methanolic T. polium extract to determine their binding modes from one side and from another site of the active residues of human peroxiredoxin 5. Recently, with the same therapeutic target, it was reported that substituted pyrazolone and dipyrazolotriazine derivatives are linked to the same active side residues of 1HD2 as those that were found in this work [53]. De Clercq et al. [54] showed that one side of the active site pocket contains several hydrophobic residues, including Leu116, Ile119, and Phe120, whose side chains are located near the benzoate aromatic ring, which can act as a hydroxyl radical scavenger (*via* its benzoate ion).

Our docking results between *S. aureus* tyrosyl-tRNA synthetase (PDB ID, 1JIJ) and *trans*-dihydrocarvone, *trans*-carveol, and eugenol are highly consistent with those involved in the ternary structure of *S. aureus* TyrRS, showing Cys37, Gly38, Ala39, Asp40, His47, Gly49, His50, Leu70, Thr75, Gln174, Asp177, Gln190, Gly192, Asp195, and Pro222 [55]. Moreover, these interactions are in full accordance with our previous work on phytocompounds from *Piper cubeba* L. EO [55] as well as with the results obtained from docking the identified compounds from *C. gigantea* flower extract [55]. In addition, the docking study of fused pyridine derivatives and new imidazo [4,5-b]pyridine-5-thione analogues has been reported to bind to several common amino acids obtained in our work [53,56]. Our docking data for the LasR binding domain corroborate with those obtained by Eswaramoorthy et al. [57], who docked isolated carbazole alkaloids and coumarins from roots of *Clausena anisate*. The authors found that their compounds possess common residues such as the commercial anti-QS ciprofloxacin. The same trend has been observed with the docking results of cladodionen, which has been proved for its high potential QS inhibitory effect against *P. aeruginosa* PAO1 [58].

## 4. Materials and Methods

### 4.1. Plant Material Sampling and Extraction of EO

Caraway seeds were purchased from a local market in August 2021 (Mahdia, Tunisia). Botanical identification was performed by Dr. Zouhair Noumi, University of Sfax, Tunisia (Voucher No: AN-0004). Essential oil from *C. carvi* L. seeds (100 g) was extracted by hydrodistillation using a Clevenger apparatus for 4 h with distilled water (500 mL). The EO was dried over anhydrous sodium sulphate and was stored in sealed glass vials in a refrigerator at 4 °C until analysis. The yield of extraction was about 4% (4 mL/100 g of dry seeds).

### 4.2. Analysis of the Volatile Compounds

GC-MS analysis was performed with a Varian CP-3800 GC equipped with an HP-5 capillary column (30 m × 0.25 mm; coating thickness 0.25 μm) and a Varian Saturn 2000 ion trap mass detector. Analytical conditions were the following: injector and transfer line temperatures were 220 and 240 °C, respectively; oven temperature was programmed from 60 to 240 °C at 3 °C/min; carrier gas helium was 1 mL/min; 0.2 μL 10% hexane solution was injected; and the split ratio was 1:30. Identification of the constituents was based on comparisons of the retention times with those of authentic standards, comparing their Linear Retention Indices relative to the series of n-hydrocarbons, and it was conducted by computer matching against commercial libraries (NIST 98 and ADAMS 95) and a home-made library of mass spectra built up from pure substances and components of known essential oils and MS literature data. Linear retention indices were calculated using the n-alkanes series (C8–C23) using the Van den Dool and Kratz formula. Moreover, the molecular weights of all identified substances were confirmed by chromatography chemical ionization mass spectrometry (GC-MS), using MeOH as a CI-ionizing gas [42,59].

### 4.3. Biological Activities of Caraway Essential Oil

#### 4.3.1. Evaluation of Anti-Vibrio spp. Activities

The antimicrobial activity of caraway essential oil was tested against a large collection of *Vibrio* spp. strains, including 17 type strains, 13 *Vibrio* spp. strains isolated from seawater, fish, and shellfish products, and 1 *Aeromonas hydrophila* ATCC 7966T strain. These microorganisms were previously isolated from diseased *Sparus aurata*, *Dicentrarchus labrax*, and *Mytilus edulis* in Tunisia [28], and the type strains were kindly provided by Professor Stefania Zanetti from the Department of Biomedical Sciences (University of Sassari, Sassari, Italy), Professor Jesús López Romalde from the Department of Microbiology and Parasitology (CIBUS-Facultad de Biologia, Universidad de Santiago, Santiago de Compostela, Spain), Professor Donatela Ottaviani from the Italian Reference Center for Microbiological and Chemical Control on Shellfish-State Veterinary Institute for Umbria and the Marches (IZSUM, Ancona, Italy), Professor Miguel Angel Morinigo from the Department of Microbiology (Facultad de ciencia de Malaga, Campus de Teatinos, Spain), and Professor Bruno Gomez Gil (Mazatlán Unit for Aquaculture, Sinaloa, Mexico).

Two techniques were used: (i) disk diffusion assay for the determination of the diameters of growth of inhibition zones estimated on Mueller–Hinton agar medium, and (ii) microdilution assay for the determination of the minimal inhibitory concentrations (MICs) and the minimal bactericidal concentrations (MBCs) [35,60]. *Vibrio* strains were grown on a Mueller–Hinton agar medium supplemented with 1% NaCl from culture stock, and pure colonies were used to prepare 0.5 McFarland turbidity. A cotton swab was used to inoculate fresh Petri dishes. Sterile filter paper disks (6 mm in diameter, Biolife, Italy) were impregnated with 10 mg of caraway EO (10.81 µL/disc) and then placed on the cultured plates. The treated Petri dishes were kept for 1 h at 4 °C and then incubated overnight at 37 °C. The diameters of growth of inhibition zones around the disks were estimated using a 1 cm flat ruler. Five antibiotics (C: chloramphenicol 30 µg; AM: ampicillin 10 µg; E: erythromycin 10 µg; TE: tetracycline 5 µg; and G: gentamycin 10 µg) were used as standard drugs against the tested *Vibrio* strains.

For the microdilution method, twofold serial dilution of the EO in DMSO-5% was prepared in 96-well plates, starting from 25 µL/mL (23.125 mg/mL) in Mueller–Hinton broth medium with 1% NaCl. A total of 5 µL of microbial inoculum was added to each well containing 100 µL of the serially diluted caraway essential oil. All microtiter plates were incubated overnight at 37 °C. MICs were defined as the lowest concentrations that are able to inhibit the growth of a specific microorganism. To determine MBC values, 3 µL from all the wells with no visible growth were point-inoculated on a Mueller–Hinton agar medium (1% NaCl). After 24 h of incubation, the concentration at which the *Vibrio* spp. strain with no growth was recorded as the MBC value.

#### 4.3.2. Evaluation of Antioxidant Activities

Antioxidant activity experiments were carried out by using four different assays: DPPH tests as described by Mseddi et al. (2020) [22], β-Carotene bleaching tests, and reducing/chelating power by using the protocols previously described [61,62,63].

For the DPPH assay, 0.25 mL of a 0.2 mM DPPH• methanolic solution was mixed with 1 mL of essential oil at different concentrations (5, 10, 15, and 20 mg/mL) or with 1 mL of control sample. The mixture was left for 30 min at room temperature in the dark. The absorbance was measured at 515 nm, and the scavenging activity (SA%) against DPPH radicals was calculated using the following Equation (1):

SA% = [(Ac − As)/Ac] × 100(1)

where Ac is the absorbance of the control at 30 min and As is the absorbance of the sample at 30 min. IC_50_ values represent the essential oil scavenging 50% of DPPH radicals. All samples were analyzed in triplicate.

For ferrous ion chelating activity, different concentrations of essential oil (1, 5, and 15 mg/mL) were added to 0.05 mL of 2 mM FeCl_2_·4H_2_O solution and were left for incubation at room temperature for 5 min. Afterwards, the reaction was initiated by adding 0.1 mL of 5 mM ferrozine, and the mixture was adjusted to 3 mL with deionized water, shaken vigorously, and left standing at room temperature for 10 min. The absorbance of the solution was then measured at 562 nm. The percentage of inhibition of ferrozine–Fe^2+^ complex formation was calculated using the following Equation (2):

Metal chelating activity (%) = [(Ac − As)/Ac] × 100(2)

where Ac is the absorbance of the control and As is the absorbance of the sample. Results are expressed as IC_50_. The IC_50_ values are the concentrations required to chelate 50% of ferrous ions present in the system. Analyses were run in triplicate.

For the reducing power assay, 1 mL of caraway essential oil was (1, 5, 10 mg/mL) mixed with 2.5 mL of phosphate buffer (0.2 M, pH 6.6) and 2.5 mL of K_3_Fe(CN)_6_ solution (1 g/100 mL). The mixture was incubated at 50 °C for 25 min, 2.5 mL of a trichloroacetic acid solution (10 g/100 mL) was added, and the mixture was centrifuged for 10 min at 650× *g*. Finally, 2.5 mL of the upper layer was mixed with 2.5 mL of distilled water and 0.5 mL of FeCl_3_ aqueous solution (0.1 g/100 mL). The absorbance of the mixture was measured at 700 nm.

The EC_50_ value (mg/mL) is the effective concentration at which the absorbance was 0.5 for the reducing power. Ascorbic acid was used as a positive control.

For the linoleic acid system, 0.2 mg of β-carotene was dissolved in 2 mL of chloroform and was added to 20 mg of linoleic acid and 200 mg of Tween 40. After removing CHCl_3_ under a vacuum, oxygenated water (100 mL) was added, and the flask was vigorously shaken until all material dissolved. The emulsion obtained was freshly prepared before each experiment. An aliquot of 150 μL of emulsion was distributed into each of the wells of 96-well microtiter plates, and 10 mg of essential oil or BHA standard solution was added. An equal amount of emulsion was used for the blank sample. The microtiter plate was incubated at 45 °C, and the absorbance was measured at 490 nm using a visible/UV microplate kinetics reader (EL × 808, Bio-Tek instruments, Winooski, VT, USA). Readings of all samples were performed immediately (t = 0 min) and after 120 min of incubation. The antioxidant activity (AA) of the essential oil was evaluated in terms of β-carotene blanching using the following Equation (3):

AA% = [(A0 − At)/A0] × 100(3)

where A0 is the absorbance of the control at 0 min and At is the absorbance of the sample (essential oil or BHA) at 120 min. The results are expressed as IC_50_ values (mg/mL).

### 4.4. Screening for Anti-Quorum Sensing Activities

#### 4.4.1. Effect on Violacein Production

*Chromobacterium violaceum* strain ATCC 12472, *C. violaceum* CV026, and *P. aeruginosa* PAO1 biosensor strains were selected to study the effects of caraway essential oil against some virulence traits controlled by the Quorum-Sensing System in both Gram-negative bacteria [40,42]. In fact, *C. violaceum* ATCC 12472 was used in qualitative screening by using the protocol previously described by Noumi et al. [64].

For the inhibition of violacein pigment production on agar media, caraway essential oil and its main compound (carvone) were loaded at 2 mg/disc on the surface of CV026-inoculated Lauria–Bertani agar plates supplemented with C_6_-HSL (50 µL of 1 mg/mL stock). The zone of violacein inhibition was detected by the presence of colourless but viable cells around the disks, and the zone of growth inhibition was also recorded by clear zones around the disks.

For violacein inhibition using a microtiter plate assay, 10 µL of *C. violaceum* ATCC 12472 was added into wells of sterile microtiter plates containing Lauria–Bertani broth. It was then incubated at 30 °C for 18 h in the presence and absence of various concentrations of caraway essential oil, ranging from 0.3125 mg/mL to 10 mg/mL at 30 °C for 18 h, and it was observed for inhibition of violacein production. For quantification of violacein, the contents of the wells were aspirated into Eppendorf tubes and centrifuged (8000 rpm 6 min) to collect cells. Violacein was extracted from the cells using water-saturated n-butanol. The extracted violacein was separated from the cell debris by centrifugation and quantified by recording OD_585_ readings spectrophotometrically. Percentage inhibition of violacein by the essential oil was calculated with respect to the control, and 50% inhibition concentration (IC_50_) was recorded.

#### 4.4.2. Effect on QS-Controlled Virulence Factor Production in *P. aeruginosa* PAO1

The effects of caraway essential oil on some factors were regulated by the QS system, including swarming motility and proteolytic and elastolytic activities, by using the protocol previously described [42].

In the swarming motility test, overnight cultures of a *P. aeruginosa* PAO1 strain were point inoculated at the center of semi-solid agar media (1% peptone, 0.5% NaCl, 0.5% agar, and 0.5% of filter-sterilized D-glucose) containing different concentrations of caraway essential oil (0.05, 0.5, 0.625, 1.25, and 2.5 mg/mL). The plate without the essential oil was used as a control. Swarming migration was recorded by following swarm fronts of bacterial cells and is expressed in mm.

For its effect on elastolytic activity, *P. aeruginosa* PAO1 was cultivated at 37 °C for 16 h in Lauria–Bertani broth media supplemented with different concentrations of caraway essential oil (0.05, 0.5, 0.625, 1.25, and 2.5 mg/mL). For the experiment, 100 μL of each concentration was mixed with 900 μL of elastin Congo red (ECR) buffer (100 mM Tris, 1 mM CaCl_2_, pH 7.5) containing 20 mg of ECR (Sigma) and was incubated for 3 h at 37 °C. After centrifugation, 200 μL of the supernatant was transferred to sterile 96-well plates, and the optical density was estimated at 495 nm.

To estimate the effects of caraway essential oil on proteolytic activity in a PAO1 starter strain, 100 μL of the bacterial culture was mixed with 900 μL of a buffer containing 3 mg of azocasein (Sigma). Eppendorf tubes were than incubated for 30 min at 37 °C.

100 μL of trichloroacetic acid (TCA, 10%) were added to each tube, and reactions were kept for 30 min. After centrifugation, the optical density of 200 μL of the supernatant was estimated at 440 nm. 

### 4.5. In Silico Approach

#### 4.5.1. Ligand Preparation

Three-dimensional structures of the ligands were retrieved through PubChem (https://pubchem.ncbi.nlm.nih.gov (accessed on 15 December 2021)) chemical information resources [65]. All the ligands were energy minimized using Avogadro, an advanced molecule editor and visualizer [66]. All the minimized ligands were converted to pdbqt before the docking procedure.

#### 4.5.2. Protein Preparation

The receptor proteins (PDB ID: 1HD2, 1JIJ, 2UV0, 2XCT, 2QP1, 3IX3, 3QPR, and 3HIR) were selected from the RSCB protein data bank (http://www.rcsb.org/ (accessed on 15 December 2021)). Water molecules and co-crystal ligands were removed from each of the proteins. The proteins were assigned polar hydrogens, Kollman charges, solvation parameters, and fragmental volumes via the Graphical User Interface program AutoDock Tools (ADT) to prepare pdbqt files. The grid around each protein was created around the binding pocket using ADT [67,68]. AutoGrid was used to create a grid map using a grid box. The grid size and grid dimensions were set for each protein according to the binding pockets, as shown in Table 8. AutoDock Vina was used to dock proteins and ligands utilizing the grid box attributes defined in the configuration file. Proteins were set as rigid throughout the docking operation, and ligands were labeled flexible. Findings with a positional root-mean-square deviation (RMSD) of less than 1.0 were grouped together and represented by the result with the lowest binding free energy. For intra-molecular interaction analysis, the pose with the lowest binding energy or affinity was selected and aligned with the receptor structure [69]. Interaction analysis was conducted by the ‘View Interaction’ protocol from the Discovery Studio (DS) program [Dassault Systems, BIOVIA Corp., San Diego, CA, USA, v 20.1].

### 4.6. ADMET Predicted Properties

The ADMET profiles of the top major identified compounds were predicted using the admetSAR online server (http://lmmd.ecust.edu.cn:8000/ (accessed on 15 December 2021)). The admetSAR server provides a user-friendly interface to easily search for chemical profiles, by CASRN, and common names and similarity searches with more than 40 predictive models were implemented in admetSAR for new chemical ADMET properties for in silico filtering.

### 4.7. Statistical Analysis

Average values of three replicates were calculated using the SPSS 25.0 (SPSS Inc., Chicago, IL, USA) statistical package for Windows. Differences in means were calculated using Duncan’s multiple-range test for means with a 95% confidence interval (*p* ≤ 0.05).

## 5. Conclusions

Overall, we report in this paper the identification of carvone and limonene as main compounds in *C. carvi* essential oil. This EO exhibits potent activity against several pathogenic and non-pathogenic *Vibrio* strains frequently isolated from fish and shellfish products with high diameters of growth inhibition zones and low minimal inhibitory concentrations for almost all tested strains. Caraway was particularly able to regulate the production of several virulence-related factors in *P. aeruginosa* PAO1 and *C. violaceum* biosensor strains. In fact, this EO was able to inhibit and eradicate biofilms formed by *V. alginolyticus*, *V. parahaemolyticus*, *V. vulnificus,* and *V. cholerae* species at sub-MIC concentrations. At 2.5 mg/mL, the production of elastase and protease by *P. aeruginosa* PAO1 was reduced by 50.17%, and 77.34%, respectively. Our computational study reveals potent ADME properties and maximal binding affinities against the tested proteins. The obtained results highlight the potential use of *C. carvi* essential oil to control pathogenic bacteria belonging to the *Vibrio* genus.

## Figures and Tables

**Table 1:** Chemical composition of *Carum carvi* L. (seeds) EO. l.r.i.: Linear Retention Index.

Code	Components	l.r.i.	Percentage	MW (g·mol^−1^)	Chemical Formula
**Monoterpene hydrocarbons (39%)**					
**1**	Myrcene	993	0.4	136.238	C_10_H_16_
**2**	D-3-carene	1013	0.1	136.238	C_10_H_16_
**3**	**Limonene**	**1032**	**38.5**	136.238	C_10_H_16_
**Oxygenated monoterpenes (59.6%)**					
**4**	*trans-p*-mentha-2.8-dien-1-ol	1126	0.1	152.237	C_10_H_16_O
**5**	*cis*-limonene oxide	1136	0.1	152.230	C_10_H_16_O
**6**	*cis-p*-mentha-2.8-dien-1-ol	1139	0.1	152.230	C_10_H_16_O
**7**	*cis*-dihydrocarvone	1195	0.7	152.230	C_10_H_16_O
**8**	*trans*-dihydrocarvone	1202	0.2	152.230	C_10_H_16_O
**9**	*trans*-carveol	1219	0.1	152.230	C_10_H_16_O
**10**	*cis*-carveol	1231	0.1	152.230	C_10_H_16_O
**11**	**Carvone**	**1242**	**58.2**	150.220	C_10_H_14_O
**Phenylpropanoids (0.1%)**					
**12**	Eugenol	1358	0.1	164.200	C_10_H_12_O_2_

**Table 2:** Antioxidant activities of caraway essential oil compared to ascorbic acid and BHT. BHT: Butylated hydroxytoluene. The letters (a–c) indicate a significant difference between the different antioxidant methods according to the Duncan test (*p* < 0.05).

	DPPH IC_50_ (mg/mL)	Reducing Power EC_50_ (mg/mL)	β-Carotene IC_50_ (mg/mL)	Chelating Power IC_50_ (mg/mL)
***C. carvi* EO**	15 ± 0.23 ^a^	7.8 ± 0.01 ^c^	3.9 ± 0.025 ^a^	6.8 ± 0.05 ^b^
**Ascorbic acid**	12 ± 0.01 ^b^	25 ± 0.01 ^a^	-	-
**BHT**	11.5 ± 0.62 ^b^	23.00 ± 1.0 ^b^	4.60 ± 1.60 ^a^	-
**EDTA**	-	-	-	32.50 ± 1.32 ^a^

**Table 3:** Antimicrobial activity of the tested *Carum* essential oil evaluated by disc diffusion and microdilution assays as compared to five antibiotics. GIZ: Mean of Growth Inhibition Zone. The letters (a–o) indicate a significant difference between the different means of GIZ according to the Duncan test (*p* < 0.05). C: chloramphenicol 30 µg; AM: ampicillin 10 µg; E: erythromycin 10 µg; TE: tetracycline 5 µg; G: gentamycin 10 µg.

Microorganisms Tested	Disc Diffusion Assay						Microdilution Assay		
	(GIZ ± SD)	C	AM	E	TE	G	MIC	MBC	MBC/MIC Ratio
*V. cholerae* ATCC 9459	23.33 ± 0.58 ^l^	27	8	6	20	18	0.022	5.781	>4; Bacteriostatic
*V. cholerae*	11.67 ± 0.58 ^cd^	8	10	6	21	20	0.022	5.781	>4; Bacteriostatic
*V. vulnificus* ATCC 27962	30.33 ± 0.58 ^n^	17	7	26	24	16	0.022	23.125	>4; Bacteriostatic
*V. vulnificus S*_5_	11.33 ± 0.58 ^c^	18	7	17	13	19	0.022	5.781	>4; Bacteriostatic
*V. vulnificus V*_30_	13.33 ± 0.58 ^e^	30	6	6	25	20	0.022	11.562	>4; Bacteriostatic
*V. parahaemolyticus* ATCC 17802	16.00 ± 0.00 ^g^	8	7	7	17	17	0.022	11.562	>4; Bacteriostatic
*V. parahaemolyticus* ATCC 43996	22.66 ± 0.58 ^l^	22	7	14	17	18	0.022	2.890	>4; Bacteriostatic
*V. parahaemolyticus* I_12_	16.66 ± 0.58 ^gh^	7	7	14	20	16	0.022	11.562	>4; Bacteriostatic
*V. parahaemolyticus* I_22_	25.33 ± 0.58 ^m^	19	10	14	15	20	0.022	11.562	>4; Bacteriostatic
*V. parahaemolyticus*	12.66 ± 0.58 ^de^	24	7	12	14	18	0.022	5.781	>4; Bacteriostatic
*V. parahaemolyticus S*_949_	11.33 ± 0.58 ^fg^	13	7	7	13	20	0.022	5.781	>4; Bacteriostatic
*V. parahaemolyticus S*_950_	11.67 ± 0.58 ^cd^	13	10	7	15	17	0.022	11.562	>4; Bacteriostatic
*V. alginolyticus* ATCC 33787	17.66 ± 0.58 ^h^	22	6	22	17	17	0.022	11.562	>4; Bacteriostatic
*V. alginolyticus* ATCC 17749	37.33 ± 0.58 ^o^	25	7	7	16	12	0.045	2.890	>4; Bacteriostatic
*V. alginolyticus*	24.66 ± 0.58 ^m^	20	6	20	15	10	0.022	11.562	>4; Bacteriostatic
*V. alginolyticus S*_6_	11.67 ± 0.58 ^cd^	13	6	7	11	12	0.022	2.890	>4; Bacteriostatic
*V. alginolyticus S*_7_	12.67 ± 0.58 ^de^	29	15	14	7	23	0.090	23.125	>4; Bacteriostatic
*V. alginolyticus S*_5_	22.66 ± 0.58 ^l^	18	8	15	13	20	0.022	2.891	>4; Bacteriostatic
*V. furnisii* ATCC 35016	8.66 ± 0.58 ^a^	33	7	30	20	13	0.022	23.125	>4; Bacteriostatic
*V. cincinnatiensis* ATCC 35912	10.33 ± 058 ^b^	24	7	30	18	15	0.022	23.125	>4; Bacteriostatic
*V. proteolyticus* ATCC 15338	20.33 ± 0.58 ^j^	24	8	10	16	16	0.022	11.562	>4; Bacteriostatic
*V. natrigens* ATCC 14048	21.67 ± 0.58 ^k^	18	13	25	16	14	0.022	5.781	>4; Bacteriostatic
*V. mimicus* ATCC 33653	21.33 ± 0.58 ^k^	18	10	12	15	14	0.022	11.562	>4; Bacteriostatic
*V. fluvialis* ATCC 33809	14.66 ± 0.58 ^f^	11	7	7	17	18	0.022	11.562	>4; Bacteriostatic
*V. anguillarum*	23.66 ± 0.58 ^l^	24	6	19	14	20	0.045	5.781	>4; Bacteriostatic
*V. carhiaccae* ATCC 35084	14.33 ± 0.58 ^f^	6	10	7	15	17	0.022	5.781	>4; Bacteriostatic
*V. harveyi* ATCC 18293	17.66 ± 0.58 ^h^	27	7	12	16	20	0.022	5.781	>4; Bacteriostatic
*V. diazotrophicus* ATCC 33466	12.00 ± 0.00 ^cd^	22	8	30	19	18	0.022	11.562	>4; Bacteriostatic
*V. tapetis* CECT 4600^T^	20.00 ± 1.00 ^ij^	10	19	6	34	15	0.022	11.562	>4; Bacteriostatic
*V. splendidus* ATCC 33125	19.33 ± 0.58 ^i^	22	6	22	24	16	0.022	11.562	>4; Bacteriostatic
*A. hydrophila* ATCC 7966^T^	16.66 ± 0.58 ^gh^	23	8	7	19	15	0.022	11.562	>4; Bacteriostatic

**Table 4:** Effect of caraway essential oil and its major compound (carvone) on the swarming activity of *P. aeruginosa* PAO1 strain expressed as mean diameter of growth on LB-0.5% agar (mm).

	Control (PAO1 Strain)	Concentrations Tested (mg/mL)				
		2.5	1.25	0.625	0.5	0.05
***C. carvi*** **EO**	54.00 ± 0.00	11.33 ± 0.57	13.33 ± 0.57	15.66 ± 0.57	16.00 ± 0.00	17.33 ± 1.15
**Carvone**	54.00 ± 0.00	11.00 ± 0.00	11.66 ± 0.57	13.00 ± 0.00	14.33 ± 0.57	15.66 ± 0.57

**Table 5:** Inhibition of violacein production by *C. violaceum* ATCC 12472 by caraway essential oil at different MIC values. The letters (a,b) indicate significant difference according to the Duncan test (*p* < 0.05).

Concentration Tested	% of Violacein Inhibition
MIC; (10 mg/mL)	47.57 ± 3.7 ^a^
MIC/2; (5 mg/mL)	32.26 ± 2.2 ^b^
MIC/4; (2.5 mg/mL)	29.17 ± 1.3 ^b^
MIC/8; (1.25 mg/mL)	28.21 ± 6.1 ^b^
MIC/16; (0.625 mg/mL)	27.89 ± 6.0 ^b^
MIC/32; (0.312 mg/mL)	25.28 ± 4.3 ^b^

**Table 6:** Best phytoconstituents with the lowest binding energies and their interaction residues with selected target proteins.

Compounds	Interacting Residues Receptor vs. Targets	Binding Energy (kcal/mol)
*trans-p*-mentha-2.8-dien-1-ol vs. 1HD2	**van der Waals:** Thr147. **Alkyl/Pi-Alkyl:** Pro40 (4.13), Pro45 (5.04), Phe120 (5.20).	−5.2
*trans*-dihydrocarvone vs. IJIJ	**van der Waals:** Ala39. **C-H bond:** Cys37(3.06), Gly38 (2.50), **Alkyl/Pi-Alkyl:** Tyr36 (4.33), Cys37 (4.49).	−6.3
*trans*-carveol vs. 1JIJ	**Alkyl/Pi-Alkyl:** Cys37 (), Ala39 (), Tyr36 ().	−6.4
Eugenol vs. IJIJ	**van der Waals:** Tyr36. **H bond:** Asp40 (2.50), Thr75 (1.95), Tyr170 (2.88). **C-H bond:** Gln196 (2.52). **Alkyl/Pi-Alkyl:** Cys37(4.50), Leu70 (5.40).	−6.3
*cis*-carveol vs. 2XCT	**C-H bond:** Leu1348; **Alkyl/Pi-Alkyl:** Ala1351, Ile1346, Leu1168, Met1335, Leu1348, Leu1167, Ile1183, Ile1346, Val1038, Tyr1234.	−5.3
Carvone vs. 2XCT	**H bond:** Ala1337 (2.55), Ile1346 (2.12). **C-H bond:** Ile1336, Leu1345. **Alkyl:** Val1038, Ala1337, Met1335, Ile1346, Leu1168, Val1339, Leu1167.	−5.3
Limonene vs. 2UV0	**van der Waals:** Val76, **Alkyl/Pi-Alkyl:** Tyr93 (5.50), Tyr56 (5.08) (5.38), Tyr64 (3.98) (4.98), Leu36 (4.82), Leu110 (3.07) (5.26) (5.42), Trp88 (3.96) (4.75).	−7.4
*trans*-carveol vs. 2UV0	**Alkyl/Pi-Alkyl:** Cys79 (5.17), Tyr47 (5.45), Val76 4.39, Leu40 (4.20), Leu125 (3.65) (3.93), Tyr64 (4.93).	−7.5
Carvone vs. 3IX3	**H bond:** Arg61 (2.71). **Alkyl/Pi-Alkyl:** Leu36 (4.67) (5.27), Tyr56 (4.94), Tyr64 (4.33) (4.76), Trp88 (5.74) (5.86). **P-Lone Pair:** Tyr64 (2.71)	−7.5
*trans*-dihydrocarvone vs. 3IX3	**van der Waals:** Tyr56. **H bond:** Trp60 (2.65), **Alkyl/Pi-Alkyl:** Leu36 (4.75), Tyr64 (4.56), Trp88 (4.56) (5.71), Leu110 (4.38).	−7.5
Limonene vs. 3QP1	**van der Waals:** Trp111, **Alkyl/Pi-Alkyl:** Ile99 (4.42), Tyr80 (4.61), Leu57 (4.23), Leu85 (4.26), Met89 (5.21), Tyr88 (3.74) (5.21).	−7.4
Carvone vs. 3QP1	**H bond:** Trp84 (3.21). **Pi-Sigma:** Trp111 (3.53). **Alkyl/Pi-Alkyl:** Leu100 (5.05), Tyr80 (4.98), Leu57 (4.29) (4.49), Leu85 (5.17), Tyr88 (4.16), Ile99 (4.37) (4.68), Phe115 (5.23), Trp111(5.37).	−7.5

**Table 7:** ADMET properties of the identified compounds from caraway essential oil. Compounds **1**–**12** are the same as those listed in Table 1.

ADMET Predicted Profile	1		2		3		4	
	Results	Probability	Results	Probability	Results	Probability	Results	Probability
**Absorption**								
Human Intestinal Absorption	+	0.9698	+	0.9819	+	0.9692	+	0.9795
Caco-2 Permeability	+	0.7783	+	0.7793	+	0.7994	+	0.6792
Blood–Brain Barrier	+	0.9967	+	0.9911	+	0.9962	+	0.9564
Human oral bioavailability	-	0.5286	+	0.8000	+	0.6143	-	0.6286
Subcellular localization	Nucleus	0.5972	Lysosomes	0.7499	Lysosomes	0.6471	Lysosomes	0.5661
P-glycoprotein inhibitor	-	0.9810	-	0.9663	-	0.9834	-	0.9846
P-glycoprotein substrate	-	0.9692	-	0.9250	-	0.9317	-	0.8969
**Distribution and Metabolism**								
CYP3A4 substrate	-	0.6665	-	0.5787	-	0.6282	-	0.5108
CYP2C9 substrate	-	0.8209	-	0.7890	-	0.8110	-	0.7759
CYP2D6 substrate	-	0.7550	-	0.7441	-	0.7415	-	0.7764
CYP3A4 inhibition	-	0.9747	-	0.8365	-	0.9257	-	0.7643
CYP2C9 inhibition	-	0.9178	-	0.7654	-	0.9308	-	0.8692
CYP2C19 inhibition	-	0.8849	-	0.7419	-	0.8906	-	0.8058
CYP2D6 inhibition	-	0.9341	-	0.9171	-	0.9398	-	0.9189
CYP1A2 inhibition	-	0.7790	-	0.7877	-	0.7497	-	0.8169
CYP inhibitory promiscuity	-	0.7215	-	0.7928	-	0.7657	-	0.9195
**Excretion and Toxicity**								
Carcinogenicity	+	0.5429	-	0.7571	-	0.4589	-	0.7857
Ames mutagenesis	-	0.9200	-	0.8700	-	1.0000	-	0.8600
Human ether-a-go-go inhibition	-	0.6620	-	0.4935	-	0.5586	-	0.5665
Micronuclear	-	0.9900	-	0.9200	-	1.0000	-	0.9700
Hepatotoxicity	-	0.8250	-	0.8250	-	0.8250	-	0.8000
Acute Oral Toxicity	III	0.8030	III	0.8186	III	0.9069	III	0.8083
Biodegradation	+	0.9000	+	0.5500	+	0.9500	+	0.8000
**ADMET Predicted Profile (Regression)**								
Water solubility	−3.443		−4.227		−3.937		−1.609	
Plasma protein binding	0.433		0.837		0.373		0.555	
Acute Oral Toxicity	1.66		1.5		1.856		3.839	
*Tetrahymena pyriformis*	1.193		0.252		0.113		−0.445	
**ADMET Predicted Profile**	**5**		**6**		**7**		**8**	
	**Results**	**Probability**	**Results**	**Probability**	**Results**	**Probability**	**Results**	**Probability**
**Absorption**								
Human Intestinal Absorption	+	0.9840	+	0.9795	+	0.9873	+	0.9873
Caco-2 Permeability	+	0.7132	+	0.6792	+	0.7226	+	0.7226
Blood–Brain Barrier	+	0.9743	+	0.9564	+	0.9778	+	0.9778
Human oral bioavailability	+	0.7000	-	0.6286	+	0.6286	+	0.6286
Subcellular localization	Lysosomes	0.5804	Lysosomes	0.5661	Mitochondria	0.5743	Mitochondria	0.5743
P-glycoprotein inhibitor	-	0.9840	-	0.9846	-	0.9772	-	0.9772
P-glycoprotein substrate	-	0.9223	-	0.8969	-	0.9097	-	0.9097
**Distribution and Metabolism**								
CYP3A4 substrate	+	0.5411	-	0.5108	-	0.6066	-	0.6066
CYP2C9 substrate	-	1.0000	-	0.7759	-	0.8012	-	0.8012
CYP2D6 substrate	-	0.7083	-	0.7764	-	0.7855	-	0.7855
CYP3A4 inhibition	-	0.8418	-	0.7643	-	0.9166	-	0.9166
CYP2C9 inhibition	-	0.5512	-	0.8692	-	0.9518	-	0.9518
CYP2C19 inhibition	+	0.5571	-	0.8058	-	0.8753	-	0.8753
CYP2D6 inhibition	-	0.9316	-	0.9189	-	0.9395	-	0.9395
CYP1A2 inhibition	+	0.7089	-	0.8169	-	0.7044	-	0.7044
CYP inhibitory promiscuity	-	0.8628	-	0.9195	-	0.8829	-	0.8829
**Excretion and Toxicity**								
Carcinogenicity	-	0.8857	-	0.7857	-	0.7857	-	0.7857
Ames mutagenesis	-	0.9000	-	0.8600	-	0.8200	-	0.8200
Human ether-a-go-go inhibition	-	0.5581	-	0.5665	-	0.7524	-	0.7524
Micronuclear	-	0.8100	-	0.9700	-	1.0000	-	1.0000
Hepatotoxicity	-	0.7500	-	0.8000	-	0.8500	-	0.8500
Acute Oral Toxicity	III	0.8102	III	0.8083	III	0.8245	III	0.8245
Biodegradation	+	0.6000	+	0.8000	+	0.8500	+	0.8500
**ADMET Predicted Profile (Regression)**								
Water solubility	−2.724	−1.609	−2.117	−2.117				
Plasma protein binding	0.589	0.555	0.394	0.394				
Acute Oral Toxicity	1.438	3.839	2.061	2.061				
*Tetrahymena pyriformis*	0.762	−0.445	0.236	0.236				
**ADMET Predicted Profile**	**9**		**10**		**11**		**12**	
	**Results**	**Probability**	**Results**	**Probability**	**Results**	**Probability**	**Results**	**Probability**
**Absorption**								
Human Intestinal Absorption	+	0.9859	+	0.9859	+	0.9956	+	0.9767
Caco-2 Permeability	+	0.7041	+	0.7041	+	0.7047	+	0.6118
Blood–Brain Barrier	+	0.9406	+	0.9406	+	0.9791	+	0.9260
Human oral bioavailability	+	0.5714	+	0.5714	+	0.5714	-	0.5857
Subcellular localization	Mitochondria	0.4869	Mitochondria	0.4869	Mitochondria	0.6420	Mitochondria	0.7362
P-glycoprotein inhibitor	-	0.9772	-	0.9772	-	0.9716	-	0.9765
P-glycoprotein substrate	-	0.8702	-	0.8702	-	0.9360	-	0.8741
**Distribution and Metabolism**								
CYP3A4 substrate	-	0.6433	-	0.6433	-	0.6171	+	0.5312
CYP2C9 substrate	-	0.8090	-	0.8090	-	0.8078	-	1.0000
CYP2D6 substrate	-	0.7021	-	0.7021	-	0.8631	-	0.6817
CYP3A4 inhibition	-	0.8309	-	0.8309	-	0.8964	-	0.9404
CYP2C9 inhibition	-	0.9206	-	0.9206	-	0.9425	-	0.9581
CYP2C19 inhibition	-	0.7038	-	0.7038	-	0.6994	-	0.8321
CYP2D6 inhibition	-	0.8987	-	0.8987	-	0.9069	-	0.9267
CYP1A2 inhibition	-	0.8306	-	0.8306	-	0.8146	-	0.8382
CYP inhibitory promiscuity	-	0.8346	-	0.8346	-	0.8246	-	0.9270
**Excretion and Toxicity**								
Carcinogenicity	-	0.8571	-	0.8571	-	0.6768	-	0.8429
Ames mutagenesis	-	0.9500	-	0.9500	-	0.9600	-	0.6300
Human ether-a-go-go inhibition	-	0.5592	-	0.5592	-	0.5429	-	0.5685
Micronuclear	-	0.8900	-	0.8900	-	0.8100	-	0.9700
Hepatotoxicity	-	0.8000	-	0.8000	-	0.6750	-	0.8750
Acute Oral Toxicity	III	0.8021	III	0.8021	III	0.8144	III	0.7376
Biodegradation	+	0.7750	+	0.7750	+	0.6500	-	0.5250
**ADMET Predicted Profile (Regression)**								
Water solubility	−2.258	−2.258	−1.998	−2.874				
Plasma protein binding	0.546	0.546	0.654	0.895				
Acute Oral Toxicity	1.896	1.896	1.845	3.394				
*Tetrahymena pyriformis*	0.162	0.162	0.6	0.012				

**Table 8:** Grid size and dimensions for the receptors.

Protein (PDB ID)	Grid Size (x, y, z Points)	Grid Dimension Center (x, y, z Coordinates)	Grid Spacing in Å
1HD2	40 × 40 × 40	7.089, 41.659, 34.385	0.375
1JIJ	40 × 40 × 40	−11.273, 13.817, 86.080	0.375
2UV0	40 × 40 × 40	23.998, 16.050, 80.315	0.375
2XCT	52 × 52 × 52	30.098, 32.836, 89.243	0.375
3IX3	52 × 52 × 52	14.630, −1.973, 9.690	0.375
3QP1	38 × 40 × 40	20.546, 12.912, 49.410	0.375
3QPR	126 × 126 × 126	54.057, 84.102, 205.034	0.375
3HIR	60 × 60 × 60	−26.388, 13.803, −15.845	0.375

## Data Availability

The data generated and analyzed during this study are included in this article.
